# Intrahepatic pseudoaneurysm following penetrating abdominal injury: Surgical and endovascular management of 2 complicated cases

**DOI:** 10.1016/j.ijscr.2020.05.017

**Published:** 2020-05-21

**Authors:** Laila H. AbuAleid, Khaled Elshaar, Almoaiad A. Alhazmi, Mohammed Al Sherbini, Khalid Albohiri

**Affiliations:** a Department of General Surgery, King Fahad Central Hospital, Jazan, Saudi Arabia; b Department of Interventional Radiology, King Fahad Central Hospital, Jazan, Saudi Arabia; c Department of Anesthesia, King Fahad Central Hospital, Jazan, Saudi Arabia; d Department of Thoracic Surgery, King Fahad Central Hospital, Jazan, Saudi Arabia

**Keywords:** Intrahepatic, Pseudoaneurysm, Hepatic artery, Trauma, Endovascular, Embolization

## Abstract

**Introduction:**

Post-traumatic pseudoaneurysm of the hepatic artery is a rare complication of abdominal injury. Prompt intervention is essential to avoid a life-threatening hemorrhage. We report two complicated cases of intrahepatic pseudoaneurysm (IHPA).

**Presentation of cases:**

Case 1: A victim of a bomb blast with thoracoabdominal injury presented in hypovolemic shock. Emergency laparotomy revealed actively bleeding liver lacerations, which had been successfully controlled with perihepatic packing. After 72 h, computed tomography (CT) of the abdomen showed a 3 cm × 1.8 cm IHPA of the left hepatic artery, which was treated with endovascular microcoils embolization. Postoperatively, the patient developed bile leakage and biliopleural fistula, which were managed conservatively. Case 2: A patient suffered a shotgun injury to the abdomen. In laparotomy, a grade III liver laceration was noted. The bleeding was controlled with perihepatic packing. The packs were removed 48 h later. Ten days postoperatively, the patient developed severe abdominal pain with shock, CT of the abdomen showed; 24 cm × 13 cm × 8 cm subcapsular liver hematoma. Superselective hepatic angiography showed a 1-cm IHPA of the right hepatic artery. The entry and exit points of the aneurysm were successfully embolized with two microcoils. No complications related to angioembolizaion were encountered.

**Discussion:**

IHPA following bomb blast and shotgun injury is rarely reported. Timely diagnosis is crucial.

**Conclusion:**

We advise to keep in mind the possibility of IHPA, when dealing with high-grade liver injury. CT is recommended before removal of perihepatic packs, as it may pick up a life-threatening pseudoaneurysm.

## Highlights

• Posttraumatic hepatic artery pseudoaneurysm is a rare complication of abdominal trauma but is a life-threatening one.
• Timely diagnosis is crucial to avoid life threatening complications of such pathology.
• Role of Damage control surgery & angioembolization in management of IHPA.

## 1 Introduction

The liver is one of the most frequently injured organs in abdominal trauma [1,2]. The anterior location in the abdominal cavity and fragile parenchyma with easily disrupted Glisson's capsule make this organ vulnerable to injury. Computed tomography (CT), angiography, enhanced critical care monitoring, and damage control surgery have revolutionized the management of liver trauma [3,4]. Angiography plays a vital role in the conservative management of liver injury. Extravasation of contrast seen on CT scan requires emergency angiography and angioembolization in hemodynamically stable patients. Postoperative angioembolization is also reported in damage control surgery prior to the removal of liver packs if rebleeding is suspected [5,6].

A pseudoaneurysm is a false aneurysm that develops from a leakage of an injured artery into the surrounding tissue, resulting in a high-pressure cavity with the risk of rupture [7]. A pseudoaneurysm can develop anywhere near an injured artery, but the common sites following trauma are hepatic and splenic artery branches [8].

We herein report two cases of penetrating abdominal injuries, with intrahepatic pseudoaneurysm (IHPA), which were treated initially with damage control surgery, followed by angioembolization of the IHPA. This work has been reported in line with the Surgical Case Report (SCARE) criteria [9].

## 2 Presentation of cases

### 2.1 Case 1

A 19-year old man presented to the emergency department, following exposure to a bomb blast injury. He had a patent airway, but he was tachypneic, agitated, confused, and pale. His blood pressure was 70/30 mm Hg and pulse rate 120 per minute. The right radial pulse was impalpable due to a compound fracture of the arm. There was a 4-cm wound in the right sixth intercostal space in the midclavicular line. The abdomen was tense and tender all over. Abdominal ultrasonography was positive for hemoperitoneum. A right-sided intercostal drainage tube was inserted for hemopneumothorax.

**Fig. 1:**
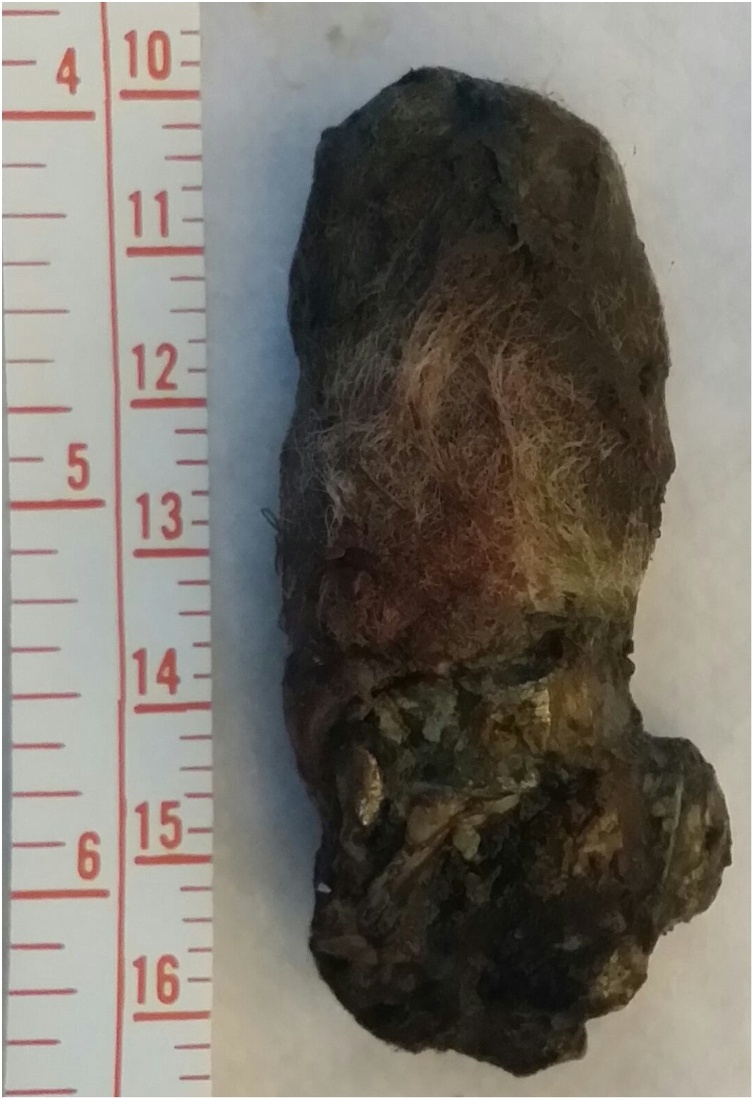
The retrieved metallic shrapnel from the abdominal cavity; 6 × 3 × 2 cm.

Exploratory laparotomy was carried out. Two liters of blood were found in the peritoneal cavity. The liver was lacerated and actively bleeding. The gallbladder was found injured; cholecystectomy was done. Suturing of the liver did not control the bleeding. Thus, a decision was made to insert perihepatic packs. Two gastric perforations were repaired. A 6 cm × 3 cm × 2 cm shrapnel was found anterior to the first part of the duodenum (Fig. 1). A drain was left near the Morrison's pouch.

The vascular surgeon explored the lacerated wound of the right arm and found that the ulnar artery was completely transected at the bifurcation of the brachial artery. Reconstruction was accomplished using a brachio-ulnar reversed saphenous vein graft.

**Fig. 2:**
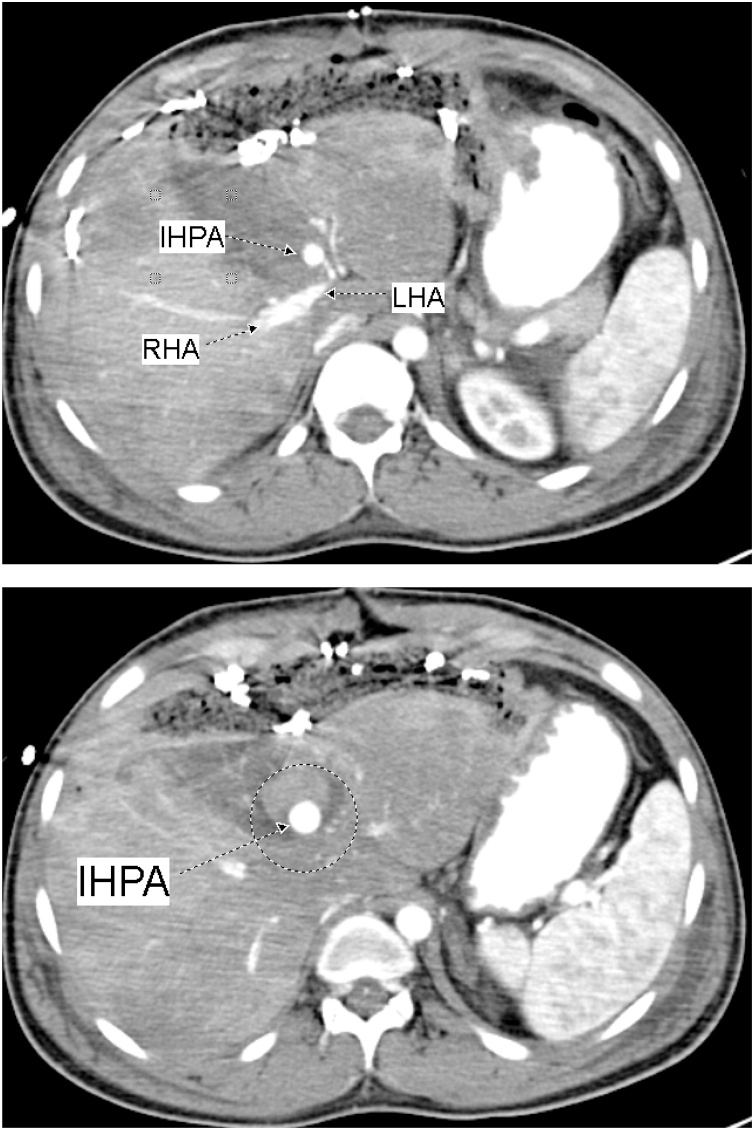
CECT Abdomen showing the Intra-Hepatic Pseudoaneurysm; IHPA, emerging from a branch of Left Hepatic Artery; LHA. The lower figure shows the IHPA surrounded by the hematoma (within the circle).

**Fig. 3:**
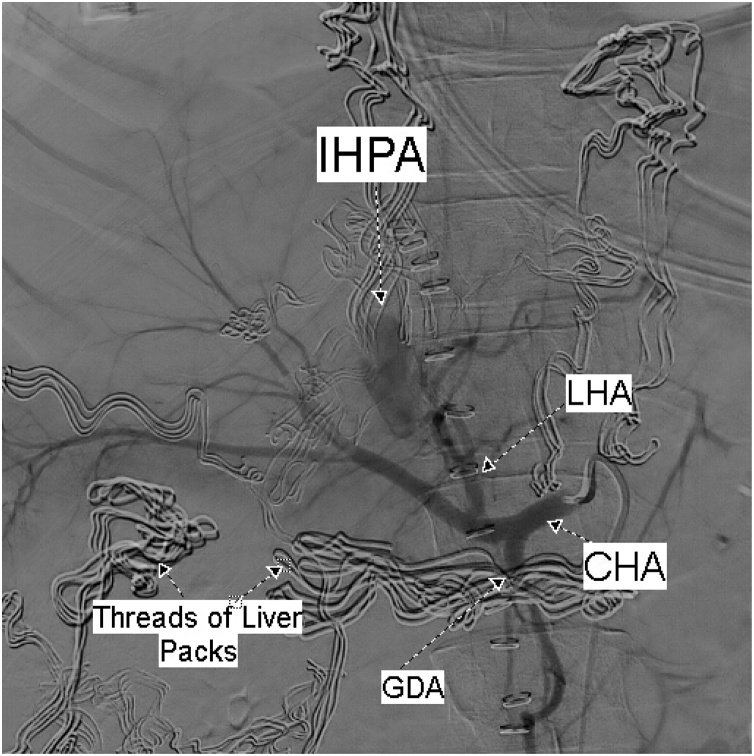
Selective celiac angiography shows the IHPA. LHA: left hepatic artery; CHA: common hepatic artery; GDA: gastroduodenal artery.

**Fig. 4:**
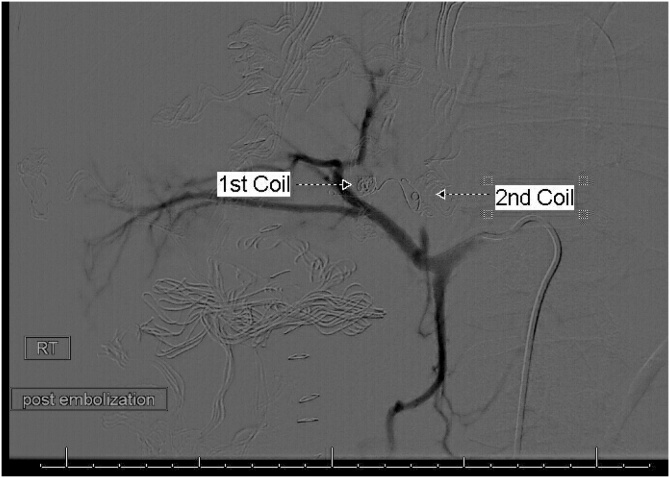
Post embolization celiac angiography shows complete exclusion of the IHPA by endo-coils.

**Fig. 5:**
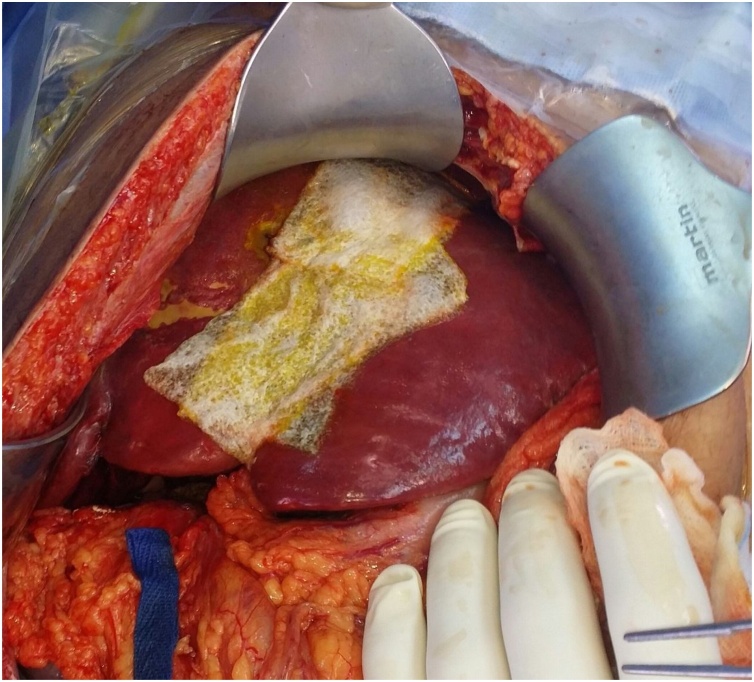
TachoSil Sealant Patches adhered well to the underlying hepatic injury, the inlet of the shrapnel.

On the first postoperative day one liter of blood was recovered from the abdominal drain. On the third postoperative day and before shifting the patient to the operating theatre for packs removal, CT of the abdomen was done; showed a 3 cm × 1.8 cm IHPA of the left hepatic artery (Fig. 2). Hepatic angiography confirmed the presence of the IHPA emerging from a side branch of the left hepatic artery (Fig. 3). Microcoil embolization of both afferent and efferent loops of the aneurysm was done (Fig. 4). The perihepatic packs were removed on the same day. There was mild oozing from the hepatic injury site, which was easily controlled with TachoSil® Fibrin Sealant Patches (Fig. 5).

**Fig. 6:**
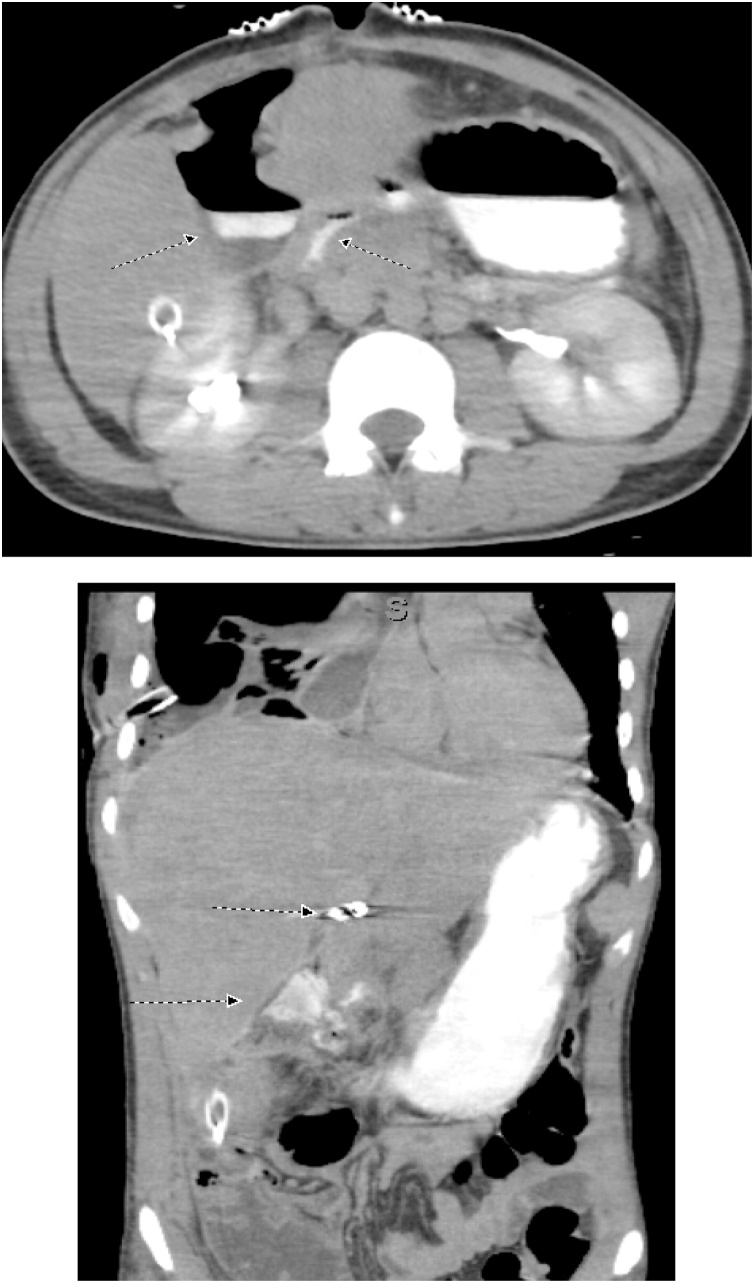
CECT Abdomen shows extra luminal leak of oral contrast from stomach to large pocket with air- fluid level in the sub-hepatic region. In the lower figure, the Intrahepatic coils seen in place, with good enhancement of both lobes of the liver.

Postoperatively, the abdominal drain started to bring bile stained fluid in large amounts, so Somatostatin Analogue, (Octreotide, Novartis Pharmaceuticals Corp), injection was started at a dose of 100 mcg, SC TID, in attempt to decrease the high output biliary fistula. On the sixth postoperative day, the patient developed upper gastrointestinal bleeding. Gastroduodenoscopy revealed mild oozing from the sutured edges of the previously repaired gastric perforations, but the procedure was complicated with an iatrogenic gastric perforation. A follow-up CT showed an extra luminal leak of the oral contrast from the stomach with a sizeable subhepatic collection, right-sided hydropneumothorax, and right lung collapse (Fig. 6). We did not notice much of gastric leak in the drain during the subsequent hours, may be because of the Octreotide effect, so we decided to keep the patient NPO, and we started Total Parenteral Nutrition (TPN), aiming to treat both gastric and biliary fistulas conservatively.

Three days later, the intercostal drainage tube started to bring bile. Hence, biliopleural fistula was diagnosed. As the patient had no significant leukocytosis and no fever at that time, we continued the conservative management with TPN and Octreotide.

**Fig. 7:**
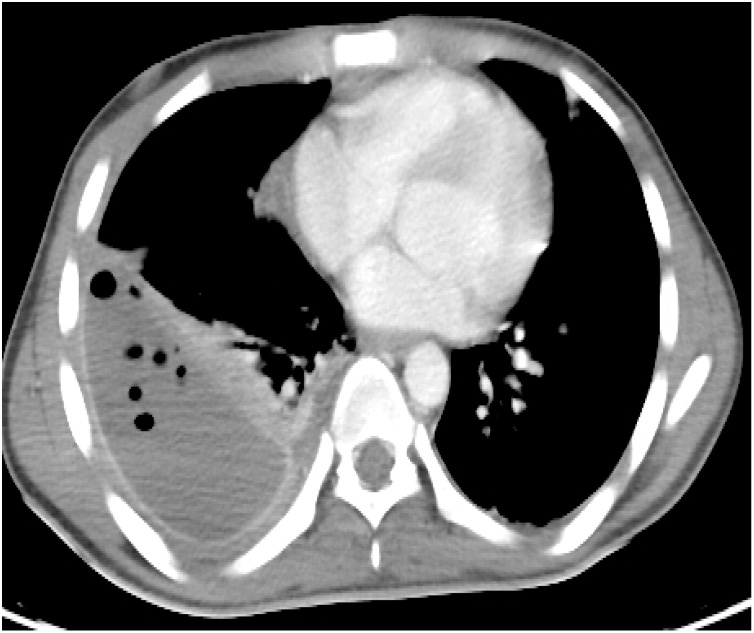
CT Chest shows the well-formed right sided empyema.

**Fig. 8:**
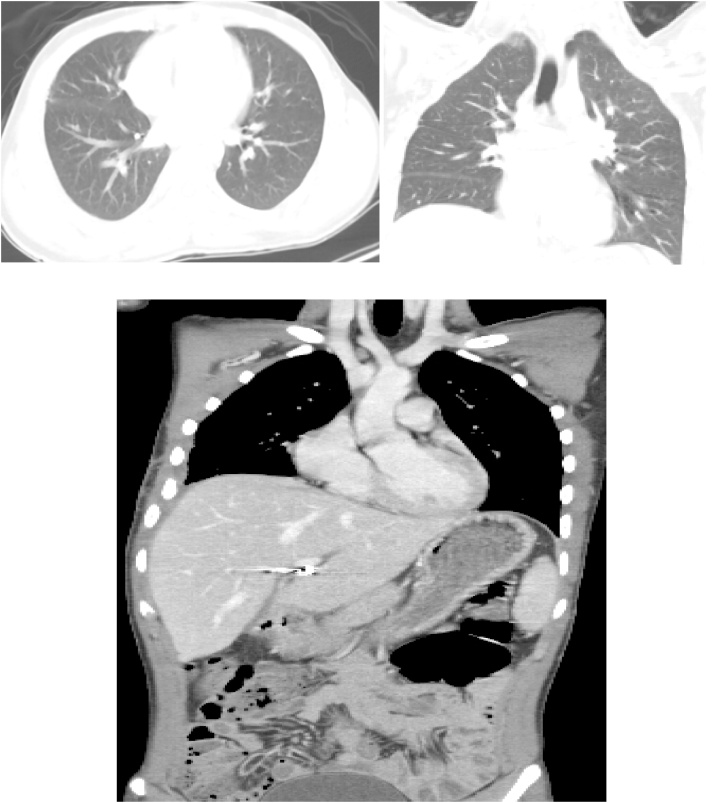
CT chest & Abdomen show normal looking both lung and liver parenchyma with no evidence of recurrence of the IHPA.

As the right-sided pleural effusion had transformed into a well-formed empyema, decortication was performed (Fig. 7). Eight weeks after admission, the patient was discharged home. He was followed up for one year; no late complications were encountered. Follow up CT showed normal looking both lung and liver parenchyma with no evidence of recurrence of the IHPA (Fig. 8).

### 2.2 Case 2

A 25-year old man was referred to the emergency department after suffering a shotgun injury of the abdomen. Apart from a congenital pectus excavatum, the chest examination was unremarkable. There was an inlet wound in the epigastrium with no exit, and the abdomen was tender all over.

At exploratory laparotomy, hemopertonium was found due to a grade III liver laceration. The bleeding could not be controlled with sutures. so we decided for perihepatic packing. Two days later, CT of the abdomen showed thrombosis of the superior mesenteric and portal veins, and there was no evidence of IHPA at that time. The packs were removed.

**Fig. 9:**
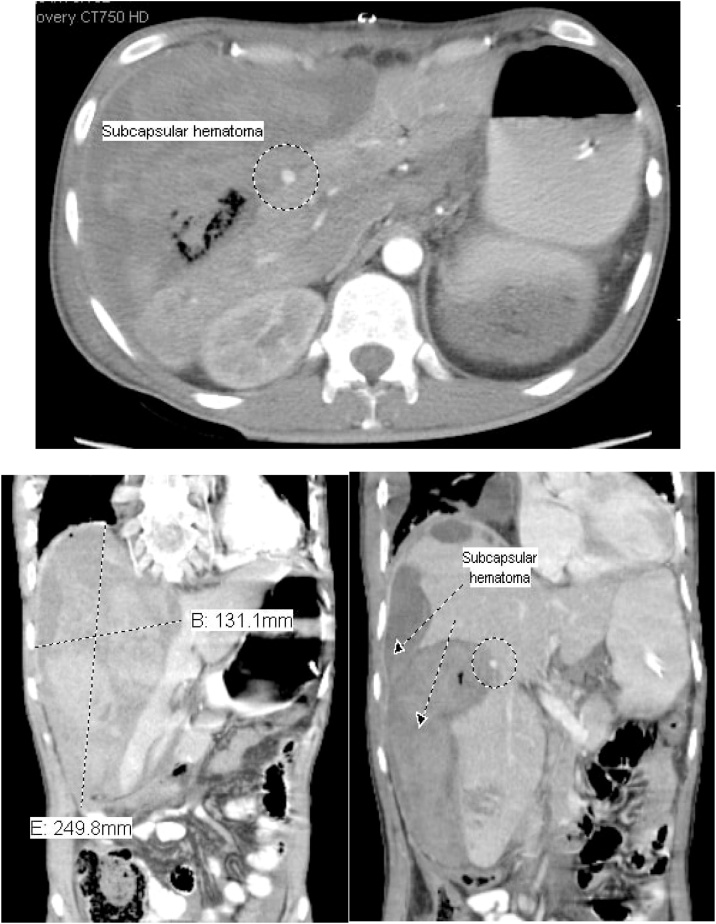
CECT abdomen showed the IHPA, marked by dotted circle along with subcapsular hematoma.

**Fig. 10:**
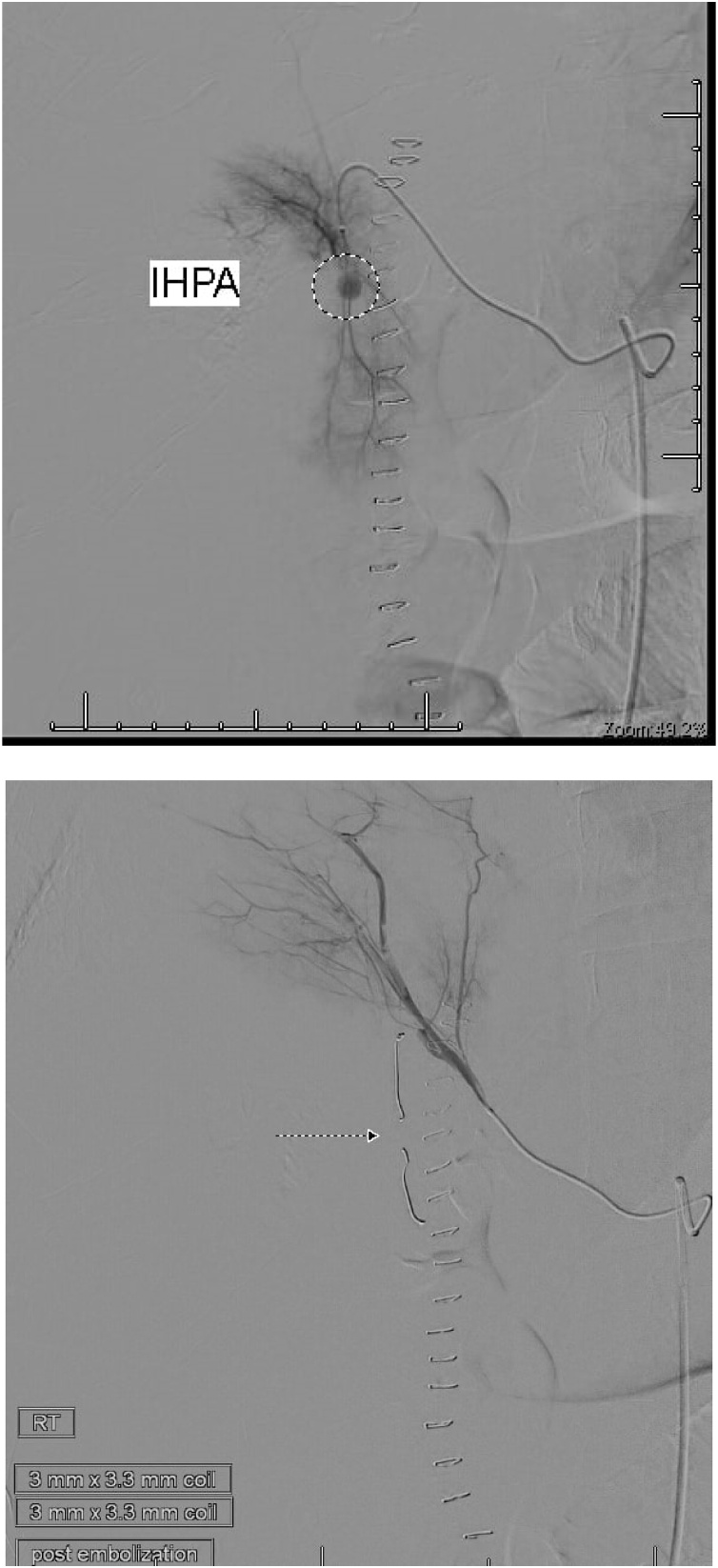
The pseudoaneurysm before embolization; upper figure, and after coils embolization; lower figure.

On the tenth postoperative day, the patient complained of a sudden severe abdominal pain. He was pale. The blood pressure was 88/48 mm Hg and pulse rate 142 per minute. The abdomen was tender. CT was done after resuscitation, showed a 24 cm × 13 cm × 8.2 cm heterogeneous subcapsular hepatic hematoma and a 1-cm IHPA (Fig. 9). Superselective hepatic angiography showed an IHPA of the right hepatic artery. The entry and exit points of the aneurysm were successfully embolized with two microcoils (Fig. 10). The patient was discharged home a few days later. No complications were reported during one year of follow up.

## 3 Discussion

Conservative treatment has become the standard management of hemodynamically stable patients with blunt liver trauma. Approximately 71%–89% of these patients are treated conservatively with a success rate of between 85%–94% [[10], [11], [12]]. CT of the abdomen is the imaging modality of choice for diagnosis and follow-up of these patients. It facilitates the evaluation of hepatic parenchyma and other abdominal and retroperitoneal organs. It also monitors the healing process and evaluates possible complications [13].

Recent literature supports operative intervention only in hemodynamically unstable patients, usually as a result of a high-velocity shotgun wound. Another indication for operative intervention is an associated hollow viscus injury [14]. Damage control surgery includes perihepatic packing and closure of the abdominal incision partially or using a Bogata bag. Kreig et al. recommend six folded laparotomy pads to be placed between the liver and the abdominal wall to obtain a tamponade effect [15]. The technique of perihepatic packing involves compressing the liver from multiple directions by placing laparotomy pads into the space between the diaphragm and liver, between the anterior and lateral abdominal walls and liver, and between the hepatic flexure of the colon and the liver. Intrahepatic packing, in which packs are placed into deep liver fractures, should not be used because it can widen the injury and lead to increased bleeding [16,17]. The patient should be transferred to the Intensive Care Unit as soon as possible for continued resuscitation and warming. As soon as the metabolic derangement is corrected, the patient should be re-explored [18]. The timing of repeat laparotomy for removal of the abdominal packs is controversial. Rates of rebleeding are higher when packs are removed less than 24 h after the initial laparotomy. However, the incidence of perihepatic sepsis is higher when a more prolonged period has elapsed. Packs removal between 24–48 hours following the initial laparotomy is a reasonable compromise [19]. In Case 1, however, we preferred to delay the removal of the packs for 72 h, as we concerned about the high possibility of rebleeding if we remove them earlier than 72 h.

Hepatic embolization appears to be most successful when used preemptively in hemodynamically stable patients who demonstrate extravasation of contrast on the initial abdominal CT. In retrospective reviews, the success rates range from 68% to 87%, and the incidence of recurrent hemorrhage is low [20]. Hepatic embolization can also be used to treat patients with failed conservative management or ongoing bleeding or rebleeding from the liver after surgical management [21].

Post-traumatic hepatic artery pseudoaneurysm is uncommon, appearing in approximately 1%–4% of hepatic trauma cases; eighty percent of the cases are extrahepatic [22,23].

IHPA may occur because of blunt or penetrating abdominal trauma. The incidence has increased along the last two decades, because of iatrogenic pseudoaneurysms resulting from the growing number of invasive procedures to manage diseases of the hepatobiliary tract. These procedures include endovascular interventions, liver biopsies, and percutaneous biliary drainage, Cholecystectomies and other gall bladder operations are responsible for up to 50% of the cases of pseudoaneurysms in this anatomical site [[24], [25], [26]].

The diagnosis of hepatic artery aneurysm is made with either conventional angiography, CT-angiography, or Doppler ultrasound [7]. On CT imaging, hepatic artery pseudoaneurysm is identified as a well-defined focal lesion of high attenuation with the same contrast enhancement as arterial structures [27]. In our cases, IHPA diagnosis was established with abdominal CT and confirmed with hepatic angiography.

Currently, endovascular management of hepatic artery pseudoaneurysm is the most common technique in hemodynamically stable patients [28]. Endoluminally inaccessible superficial pseudoaneurysms can be managed by direct percutaneous coil or thrombin embolization [29,30]. The accessible ones may be managed by pseudoaneurysm embolization or placement of a stent-graft across the artery [29,31]. As stent placement is not possible in small branches of the hepatic artery, like in our cases, coil embolization of the pseudoaneurysm was the treatment of choice.

Direct endovascular embolization of hepatic artery pseudoaneurysms with N-Butyl-Cyanoacrylate (NBCA) has excellent success rates, especially when stent or coil embolization is not possible. The main disadvantage of using NBCA is its possible passage into the distal circulation, causing hepatic necrosis in patients with compromised portal venous flow. However, this is rare in expert hands; the dual blood supply of the liver is an additional protection [28]. Coils are the most used materials. The afferent and efferent arterial segments should be embolized to avoid retrograde filling. Their success rates are 70%–100%. Occasionally, the pseudoaneurysm cannot be thrombosed this way because either the occlusion of the vessel is not complete or because it is fed by collaterals. In these situations, embolization should be attempted again through the endovascular route. Distal coil migration, hepatic abscess, and hepatic ischemia are some of the reported complications of endovascular hepatic artery embolization [32]. In our patients, however, no early or late complications occurred, as evidenced by early and delayed CT.

## 4 Conclusion

Post-traumatic hepatic artery pseudoaneurysm is a rare complication of abdominal trauma but is a life-threatening one. Timely diagnosis and treatment with endovascular intervention is essential. We advise to keep it in mind, especially when dealing with high-grade liver injury. We suggest doing CT before taking the patient to the operating theatre for hepatic packs removal, as it may pick up a possible IHPA, to avoid in-future complications of such pathology.

## Declaration of Competing Interest

No conflicts of interest.

## Source of funding

Personal funding.

## Ethical approval

The case report exempt from echical approval.

## Consent

Written informed consent was obtained from both patient for publication of this case report and accompanying images*.

## Authors contribution

Laila H AbuAleid: surgical management, data collection & manuscript writing.

Khaled Elshaar: surgical management, data collection & manuscript writing.

Almoaiad A Alhazmi: data collection & intervention radiology management.

Mohammed Al Sherbini: data collection & anathesia management.

Khalid Albohiri: thoracic surgery management & data collection.

## Registration of research studies

NA.

## Guarantor

Laila H AbuAleid.

## Provenance and peer review

Editorially reviewed, not externally peer-reviewed.
